# Hsp90 inhibition as a means to inhibit activation of the NLRP3 inflammasome

**DOI:** 10.1038/s41598-018-25123-2

**Published:** 2018-04-30

**Authors:** Niina Piippo, Eveliina Korhonen, Maria Hytti, Heli Skottman, Kati Kinnunen, Natasha Josifovska, Goran Petrovski, Kai Kaarniranta, Anu Kauppinen

**Affiliations:** 1 School of Pharmacy, University of Eastern Finland, Kuopio, 70211 Finland; 2 Faculty of Medicine and Life Sciences, BioMediTech, University of Tampere, Tampere, 33014 Finland; 3 Department of Ophthalmology, Kuopio University Hospital, Kuopio, 70211 Finland; 4 Stem Cells and Eye Research Laboratory, Department of Ophthalmology, Faculty of Medicine, Albert Szent-Györgyi Clinical Center, University of Szeged, Szeged, Hungary; 5 Center for Eye Research, Department of Ophthalmology, Oslo University Hospital, University of Oslo, Oslo, Norway; 6 Department of Ophthalmology, Institute of Clinical Medicine, University of Eastern Finland, Kuopio, 70211 Finland

## Abstract

Once activated, the intracellular receptor NLRP3 assembles an inflammasome protein complex that facilitates the caspase-1-mediated maturation of IL-1β and IL-18. Inactive NLRP3 is guarded by a protein complex containing Hsp90. In response to stress stimuli, Hsp90 is released, and NLRP3 can be activated to promote inflammation. In this study, we blocked Hsp90 with geldanamycin and studied the fate of NLRP3 in human retinal pigment epithelial (RPE) cells. RPE cells play a central role in the development of age-related macular degeneration (AMD), a progressive eye disease causing severe vision loss in the elderly. IL-1α-primed ARPE-19 cells, human embryonal stem cell (hESC)-derived RPE cells, and primary human RPE cells were exposed to MG-132 and bafilomycin A to activate NLRP3 via the inhibition of proteasomes and autophagy, respectively. Additionally, RPE cells were treated with geldanamycin at different time points and the levels of NLRP3 and IL-1β were determined. Caspase-1 activity was measured using a commercial assay. Geldanamycin prevented the activation of the inflammasome in human RPE cells. NLRP3 released from its protective complex became degraded by autophagy or secreted from the cells. Controlled destruction of NLRP3 is a potential way to regulate the inflammation associated with chronic diseases, such as AMD.

## Introduction

**Figure 1:**
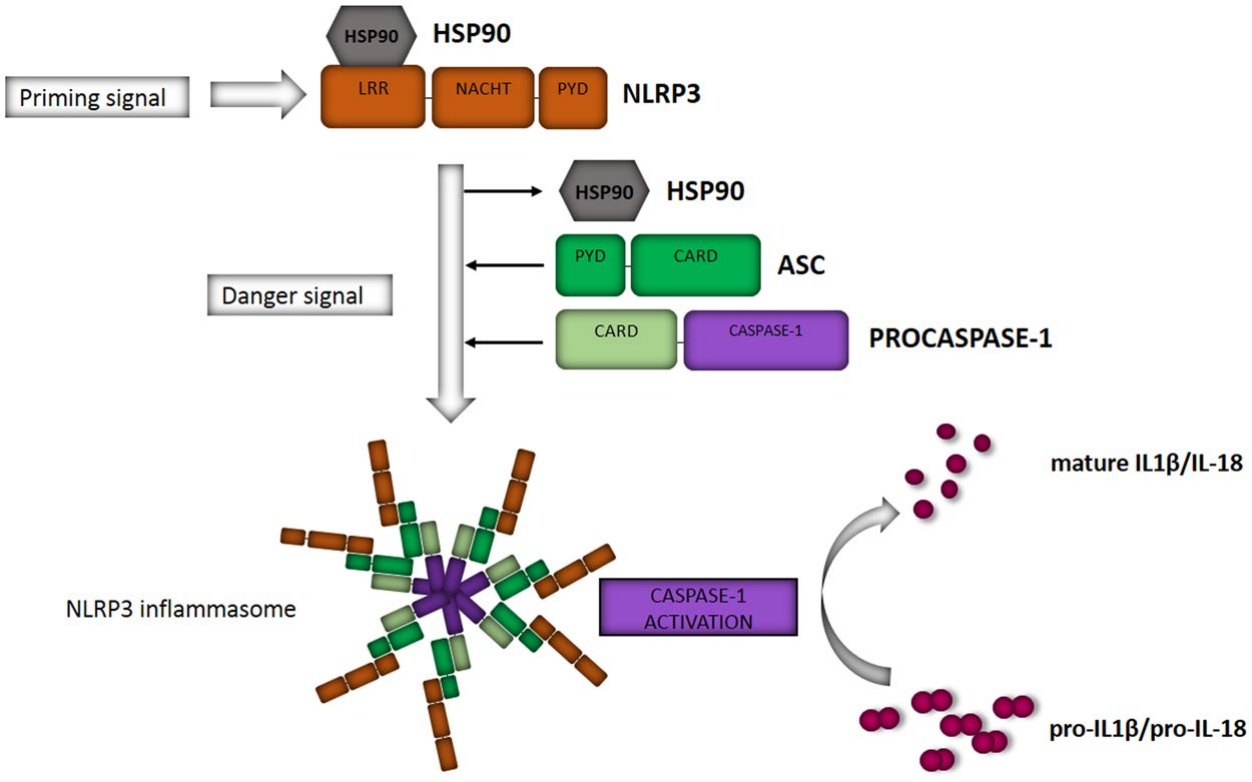
Activation of the NLRP3 inflammasome as a two-step process. After a priming signal NLRP3 protein is synthesized and protected from degradation by a protein complex containing Hsp90. After a second signal, NLRP3 is released from its chaperone, oligomerizes and recruits the receptor protein ASC and pro-Caspase-1 to form the NLRP3 inflammasome. Auto-activation of caspase-1 subsequently leads to the cleavage of pro-IL-1β and pro-IL-18 into their active forms.

The nucleotide-binding domain and Leucine-rich repeat Receptor containing a Pyrin domain 3 (NLRP3) inflammasome is an intracellular signaling complex involved in the induction of inflammation^1^. NLRP3 is a pattern-recognition receptor (PRR) that becomes activated in a two-step process. In the priming step, there is initiation of the production of NLRP3 protein and the inactive pro-form of the pro-inflammatory cytokine IL-1β e.g. via the NF-κB signaling triggered by Toll-like receptor (TLR), nucleotide-binding oligomerization domain-like receptors (NOD) receptor, or cytokine receptor activation^2,3^. Thereafter, a wide variety of danger signals of both endogenous and exogenous origins can serve as activators for NLRP3^4–6^. Activation results in the oligomerization of NLRP3 followed by the recruitment of the adaptor protein apoptosis-associated speck-like protein containing CARD (ASC) and the pro-caspase-1 into the complex, finally resulting in the auto-activation of caspase-1^1^. The activated enzyme then cleaves the pro-forms of inflammatory cytokines, IL-1β and IL-18, into their mature forms that can be secreted out of the cell (Fig. 1).

Low-level inflammation is an integral component of many chronic diseases, and activation of the NLRP3 inflammasome has recently also been associated with the pathogenesis of age-related macular degeneration (AMD)^7–9^. AMD is the leading cause of blindness among the elderly in the developed countries^10^. Prolongation of the life expectancies of the population will further increase the prevalence of AMD, emphasizing not only the personal suffering but also representing a financial, and societal health care burden^11^. New therapy options are urgently needed since there is no treatment available for the majority of patients^12^.

Heat shock protein 90 (Hsp90) is a multifunctional molecular chaperone that regulates the stability and the activation of several proteins (clients) related to signal transduction, protein trafficking, immunity, and receptor maturation^13^. NLRP3 has also emerged as one of the Hsp90 clients. In conjunction with the ubiquitin ligase-associated protein suppressor of the G2 allele of SKP1 (SGT1), Hsp90 forms a complex with NLRP3 and retains the receptor protein in an inactive but competent form for activation after reception of the priming signal^14–16^. If this complex is not formed, then the NLRP3 protein will be degraded, but it is far from clear whether the degradation takes place in proteasomes or via the lysosomal (autophagy) pathway^14,17^. In the present study, the effects of the Hsp90 inhibitor geldanamycin (GA) on the fate of NLRP3 were explored in human RPE cells.

## Methods

### Cells and stimulations

ARPE-19 cells (American Type Culture Collection (ATCC), Manassas, VA, USA) were cultured under standard conditions in a humidified 5% CO_2_ atmosphere at 37 °C in Dulbecco’s modified Eagle’s medium (DMEM) and nutrient mixture F-12 1:1 mixture (Life Technologies, Carlsbad, CA, USA) containing 10% inactivated FBS (Thermo Fisher Scientific, Waltham, MA, USA), 100 units ml^−1^ penicillin, 100 µg ml^−1^ streptomycin, and 2 mM L-glutamine (all Lonza, Basel, Switzerland). For experiments, cells were placed on 12-well plates at a concentration of 200,000 cells ml^−1^ per well in serum-containing medium, and incubated for three days. Confluent cell cultures were washed with serum-free DMEM/F12 medium and primed with recombinant IL-1α (4 ng ml^−1^, R&D Systems, Abington, UK) in serum-free medium. After 24 h incubation, the cells were exposed to MG-132 (5 µM, Calbiochem, San Diego, CA, USA) for 24 h. The cells were further stimulated with bafilomycin A1 (BafA, 50 nM) or chloroquine (CHQ, 20 µM, both, Sigma-Aldrich, Munich, Germany) for an additional 24 h. The selected concentrations are based on our previous studies^18^. Where indicated, geldanamycin (GA; 0.25 µM, Calbiochem) was added to the cell cultures just before MG-132 or BafA, or six hours after BafA. Cell culture medium as well as cell lysates were collected after incubation for 24 h as described below. GA, MG-132, and BafA were solubilized in DMSO (Sigma-Aldrich).

### Human embryonic stem cell-derived RPE cells (hESC-RPE)

The hESC line Regea08/017 (46;XX) previously derived^19^ in our laboratory at BioMediTech, University of Tampere was used in this study. The undifferentiated hESCs were cultured in the hESC culture medium consisting of KnockOut DMEM supplemented with 20% KnockOut Serum Replacement (ko-SR), 2 mM Glutamax, 0.1 mM 2-mercaptoethanol (all Thermo Fisher Scientific), 1% Non-essential amino acids, 50 U ml^−1^ penicillin/streptomycin (both Lonza), and 8 ng ml^−1^ human basic fibroblast growth factor (bFGF, Peprotech) on inactivated human foreskin fibroblast feeder cells (CRL-2429, ATCC). Undifferentiated stem cell colonies were passaged mechanically or with TrypLE Select (Thermo Fisher Scientific) onto a fresh feeder cell layer every 6 to 10 days. The hESC line is routinely karyotyped and characterized for self-renewal and differentiation capacities as well as checking for the absence of mycoplasma.

The hESCs were differentiated to RPE as previously described^20^. Briefly, hESCs were cultured as floating cell aggregates in Low Cell Binding Surface Dishes (Nunc) in RPE basic medium consisting of the same reagents as the hESC medium except that it contained 15% ko-SR but no bFGF. For enrichment, pigmented cells were isolated and seeded onto human placental collagen IV (5 µg cm^−2^, Sigma-Aldrich)-coated culture plates. The use of human embryos for research purposes at BioMediTech has been approved by the National Authority for Medicolegal Affairs Finland (Dnro 1426/32/300/05). The institute also has supportive statements from the Ethical Committee of the Pirkanmaa Hospital District to derive, culture, and differentiate hESC lines (Skottman/R05116). No new cell lines were derived for this study.

### Primary human RPE cells

Primary human RPE cells were isolated from cadavers without known ocular diseases following the Guidelines of the Declaration of Helsinki. The study was approved by the Regional Ethics Committee of the Medical and Health Science Center (IREC), University of Debrecen, Hungary (DEOEC RKEB/IKEB Prot. No. 3093-2010). The EU Directive 2004/23/EC on presumed consent practice for tissue collection is applied in Hungary and pertains to the samples collected for and used in the experiments approved by the IREC. The isolation of primary human RPE cells was started by removing the anterior segment (corneo-scleral ring) and the lens. Next, the vitreous and the neuroretina were removed using paper sponges and forceps, respectively. The RPE layer was carefully scraped using half-spherically bent-end Pasteur glass pipettes without damaging the Bruch’s membrane. The cell suspension was placed in PBS (Sigma-Aldrich) and centrifuged for 10 min at 200 × g, as described previously^21^. Cells were re-suspended in the DMEM Nutrient mixture F12 medium (Sigma-Aldrich) supplemented with 10% FBS (Gibco, Paisley, UK), 200 mM L-glutamine (Sigma-Aldrich), and 1% antibiotic/antimycotic solution (Gibcon), and incubated on cell culture plates in a humidified CO_2_ atmosphere. Experiments were performed on passage 0 confluent cultures containing 2.5 × 10^5^ cells per well. The cells were exposed to IL-1α, MG-132, and BafA using the same protocol as for ARPE-19 cells. Cell culture medium samples were collected for IL-1β release measurements and three parallel samples/group were analysed accordingly.

### Sample preparation

Medium samples and cell lysates were collected 24 h after the BafA treatment. Cells were rinsed with Dulbecco’s phosphate buffered saline (DPBS, Lonza)) before lysis with the specific buffer required for the caspase-1 activity assay. Cell lysates were centrifuged (16 060 × g, 10 min) and the supernatants were transferred into clean tubes. Protein concentrations were measured by the Bradford method from cell lysates^22^. Both medium samples and cell lysates were stored at −70 °C until analyzed.

### ELISA measurements

The extracellular concentration of the pro-inflammatory cytokine IL-1β was determined using a specific BD OptEIA^TM^ human ELISA kit (BD, Franklin Lakes, NJ, USA). Additionally, the levels of NLRP3 were measured using a commercial ELISA kit (Cusabio, Wuhan, China) according to the manufacturer’s instructions.

### Caspase-1 activity assay

The caspase-1 activity was measured from cell lysates using a commercial kit (R&D Systems) according to the manufacturer’s protocol. Results were normalized to protein concentrations.

### Western blot

For detecting NLRP3, 20 µg of protein in whole-cell lysates was mixed with 4× protein loading solution (Life Technologies) supplemented with 5% β-mercaptoethanol. Samples were run in 15% SDS–PAGE gels and wet-blotted overnight onto nitrocellulose membranes (Amersham, Piscatawy, NJ, USA). The membranes were blocked for 2 h in 5% fat-free milk in 0.1% Tween-20/Tris buffered saline (TBS; 50 mM Tris, 150 mM NaCl) at room temperature (RT). The blocking buffer without milk was also used as a washing buffer and as a diluent for the primary and secondary antibodies. Thereafter, the membranes were incubated overnight at +4 °C with a rabbit monoclonal NLRP3 antibody (1:1000, Abcam, Cambridge, UK). After washing three times for 5 min, the membranes were incubated for 2 h at RT with horseradish peroxidase-conjugated anti-rabbit IgG antibody (1:5000, Life Technologies). Before detection, the membranes were washed as described above. Protein/antibody complexes were detected with an enhanced chemiluminescent (ECL) assay for horseradish peroxidase (Millipore, Billerica, MA, USA) on Super Rx medical X-ray film (Fuji Corporation, Tokyo, Japan). The band intensities were quantified using the ImageJ software (U. S. National Institutes of Health, Bethesda, MD, USA; http://rsb.info.nih.gov/ij) and normalized to α-tubulin values using mouse monoclonal α-tubulin (Sigma-Aldrich) with polyclonal HRP-conjugated sheep anti-mouse IgG antibody (GE Healthcare, Fairfield, CT, USA).

### Data and statistical analysis

The data and statistical analysis in this study comply with the recommendations on experimental design and analysis in pharmacology^23^. Statistical analyses were conducted using GraphPad Prism 6.02 (Graphpad Software, San Diego, CA). Pairwise comparisons were performed using the Mann–Whitney *U*-test, and *P*-values of 0.05 or less were considered significant and sub-divided into three categories indicated with one, two, or three asterisks (*P < 0.05, **P < 0.01, ***P < 0.001). Each experiment was commonly repeated at least three times but depending on the experimental settings, some results in the present study were repeated up to seven times, and all data were utilized. In certain cases, the number of repetitions may have been restricted due to e.g. limited amounts of materials, such as available hESC-RPE and primary human RPE cells produced for this study.

## Results

### IL-1α efficiently primes RPE cells for the MG-132 + BafA-induced inflammasome activation

**Figure 2:**
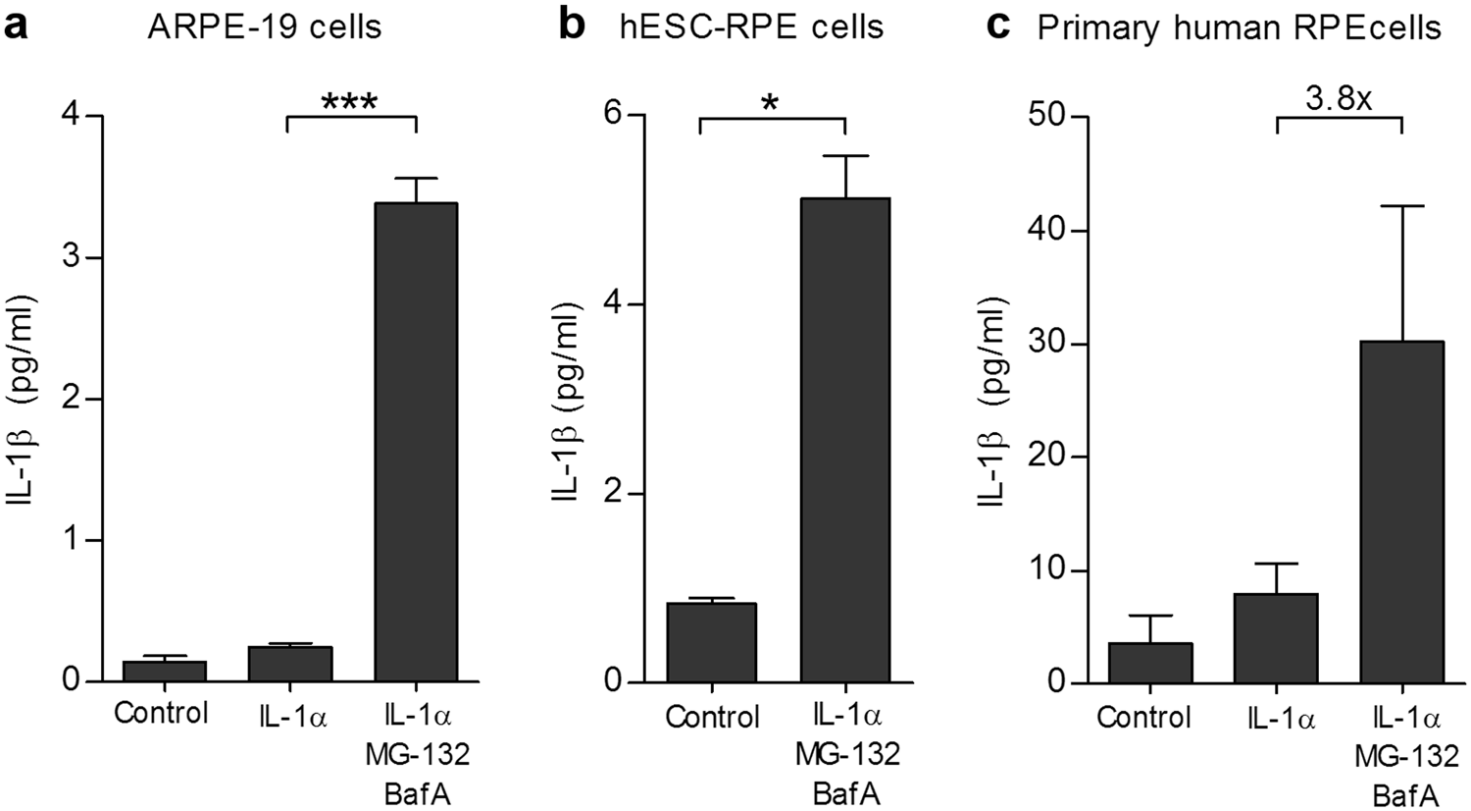
MG-132 and bafilomycin A1 (BafA) induce IL-1β secretion in IL-1α-primed ARPE-19 (**a**), hESC-RPE (**b**), and primary human RPE cells (**c**). The cells were primed *in vitro* with 4 ng ml^−1^ of IL-1α for 24 h, treated with 5 μM MG-132 for 24 h, and exposed to 50 nM BafA for an additional 24 h. Caspase-1 inhibitor (50 µM) was added prior to both MG-132 and BafA where indicated. Untreated cells plated in the same numbers and grown under similar conditions served as blank controls, and primed cells (IL-1α) served as control groups. Identical volumes of culture medium samples were taken and IL-1β was measured using the ELISA technique. Results are presented as mean ± SEM. *P < 0.05, **P < 0.01, ***P < 0.001, Mann –Whitney *U*-test to groups with at least four parallel samples.

We have previously shown that inhibition of the intracellular clearance systems with MG-132 and BafA activates NLRP3 inflammasomes, leading to the secretion of IL-1β from ARPE-19 cells^18^. Although the inflammasome was activated without any additional signal e.g. through TLR or cytokine receptors, in this study, we provided the cells with an additional priming signal using IL-1α that activates the *IL1B* promoter via the NF-κB signaling pathway^3^. IL-1α priming alone induced only 1.8 times higher IL-1β secretion when compared to untreated ARPE-19 cells (Fig. 2a). BafA triggered a 2.4-fold increase, whereas MG-132 evoked a 10.8-fold increase in the amounts of secreted IL-1β, when compared to primed cells alone (not shown in Fig. 2). When administered together, MG-132 and BafA further enhanced the IL-1β secretion to 13.7-fold when compared to primed cells (Fig. 2a). These data indicate that priming alone does not activate the inflammasome but potentiates the effects of MG-132 and BafA. In our previous study, the secreted IL-1β levels induced by MG-132 + BafA remained below 0.2 pg/ml but in the present study, the same activators on primed ARPE-19 cells raised the levels of released cytokine up to 3.4 ± 0.18 pg/ml (mean ± SEM).

In order to demonstrate that there was inflammasome activation in other RPE cells in addition to ARPE-19, hESC–RPEs and primary human RPE cells were stimulated in a similar manner. An exposure of IL-1α-primed hESC-RPE cells to MG-132 + BafA treatment significantly increased the IL-1β levels when compared to control cells (Fig. 2b; 5.1 ± 0.5 pg ml^−1^ vs. 0.8 ± 0.1 pg ml^−1^). MG-132 + BafA induced a 3.8 times higher IL-1β release also from IL-1α-pre-treated primary human RPE cells when compared to primed cells alone (Fig. 2c; 30.2 ± 12.0 pg ml^−1^ vs. 8.0 ± 2.6 pg ml^−1^). It is notable that the response of hESC-RPE cells was slightly higher than that obtained with the ARPE-19 cells (5.1 ± 0.5 vs. 3.4 ± 0.2 pg ml^−1^), while primary hRPE cells produced clearly higher levels of IL-1β when compared to ARPE-19 or hESC-RPE cells alone (30.2 ± 12.0 pg ml^−1^).

### Geldanamycin prevents the inflammasome activation in ARPE-19 and primary human RPE cells

**Figure 3:**
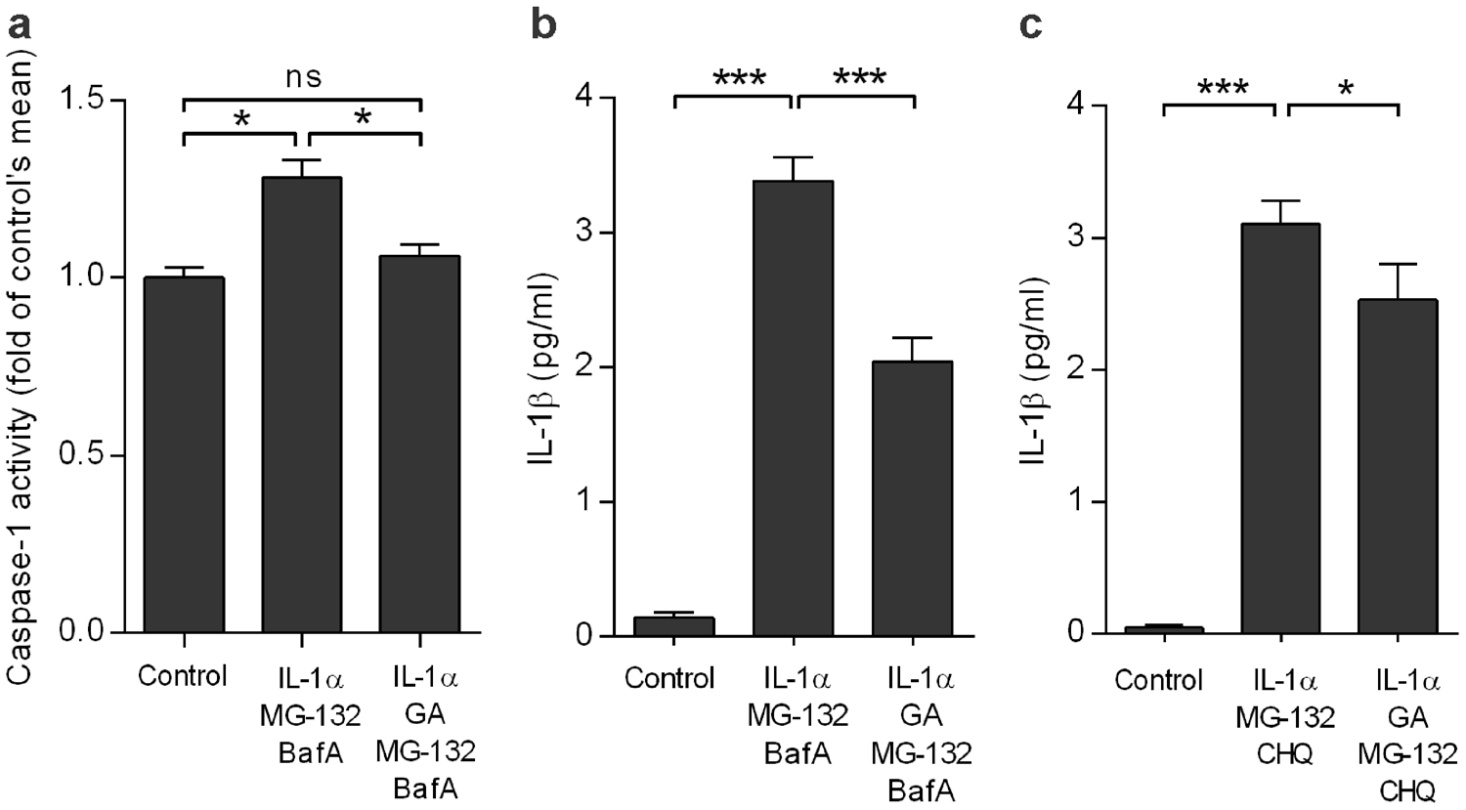
Geldanamycin (GA) inhibits the NLRP3 activation in ARPE-19 cells. Treatment with GA reduced the activity of caspase-1 (**a**) and the release of IL-1β (**b**, **c**). Caspase-1 activity was measured from the cell lysates using a commercial kit. IL-1β was measured with ELISA from the MG-132 and bafilomycin A1 (BafA) or MG-132 and chloroquine (CHQ)-treated cell culture medium samples. Results are presented as mean ± SEM. *P < 0.05, ***P < 0.001, ns = not significant, Mann–Whitney *U*-test.

Next, we studied the effects of Hsp90 inhibitor geldanamycin (GA) on the activation of the NLRP3 inflammasome. Pre-treatment of primed cells with GA resulted in decreased caspase-1 activity (Fig. 3a) and reduced IL-1β release (Fig. 3b) in ARPE-19 cells exposed to MG-132 + BafA. The finding was also verified by replacing the autophagy inhibitor BafA with chloroquine, another lysosomotropic agent that prevents the fusion of autophagosomes with lysosomes and inhibits lysosomal enzymes (Fig. 3c). Moreover, GA returned the IL-1β production of MG-132 + BafA-treated primary hRPE cells to a similar level as present in primed cells, which was comparable to the effect of treatment with the caspase-1 inhibitor (data not shown). These data provide evidence that GA is efficient on preventing the MG-132 + BafA-induced inflammasome activation in human RPE cells. The findings remained similar with different autophagy inhibitors (BafA vs. choroquine) as well as on different RPE cell models (ARPE-19 vs. primary human RPE cells).

### NLRP3 becomes degraded by autophagy

**Figure 4:**
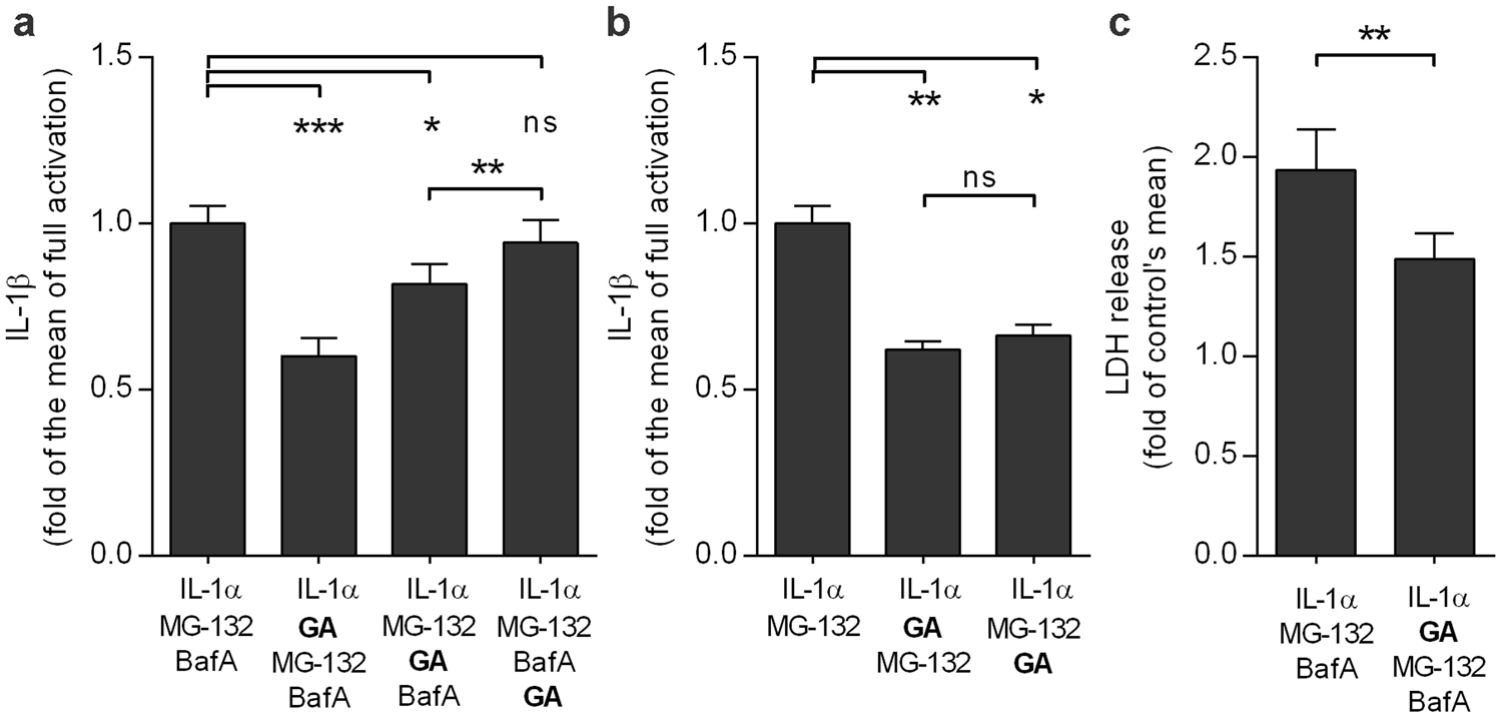
Geldanamycin (GA) efficiently prevents the IL-1β release until autophagy becomes blocked (**a**). In cells exposed only to the proteasome inhibitor MG-132 GA remains effective irrespective of treatment timepoint (**b**). The reduced release of IL-1β was not caused by increased cell death after GA addition (**c**). ARPE-19 cells were exposed to 5 μM MG-132 for 24 h and to 50 nM bafilomycin A1 (BafA) for another 24 h (**a**). GA (0.25 µM) was added prior to MG-132 (**a**,**b**), just before BafA, or six hours after BafA (**a**) or 24 h after MG-132 (**b**). IL-1β was measured from medium samples with the ELISA technique. The levels of LDH were measured from the cell culture medium using a commercial assay. Results are presented as mean ± SEM. *P < 0.05, **P < 0.01, ***P < 0.001, ns = not significant, Mann –Whitney *U*-test.

In order to study the fate of the NLRP3 released by the Hsp90 inhibitor, GA was added to cells in different stages of the activation process. The effect of GA was strongest when added immediately prior to proteasome inhibition with MG-132 (Fig. 4a). Significantly reduced IL-1β production was also detected when GA was added just before inhibition of autophagy by BafA. Conversely, the inhibitory effect of GA remained absent when it was added 6 h after BafA (Fig. 4a). In contrast, GA was capable of decreasing the secretion of IL-1β regardless of the administration time-point in cells with dysfunctional proteasome clearance after MG-132 treatment alone. (Fig. 4b). It is notable that GA-treated cells showed a significantly reduced release of LDH, indicating that the diminished release of IL-1β did not result from cell death (Fig. 4c). Together, these data suggest that NLRP3 becomes degraded by autophagy rather than in the proteasomes after its release from the Hsp90 complex in human RPE cells. This can be concluded from the data showing that the inhibitory effect of GA was visible only when autophagy was still functional (prior to the addition of BafA). Proteasomal inhibition was not enough to prevent the NLRP3 activation.

### MG-132 + BafA treatment decreases intracellular NLRP3 and increases extracellular NLRP3, while GA attenuates these effects

**Figure 5:**
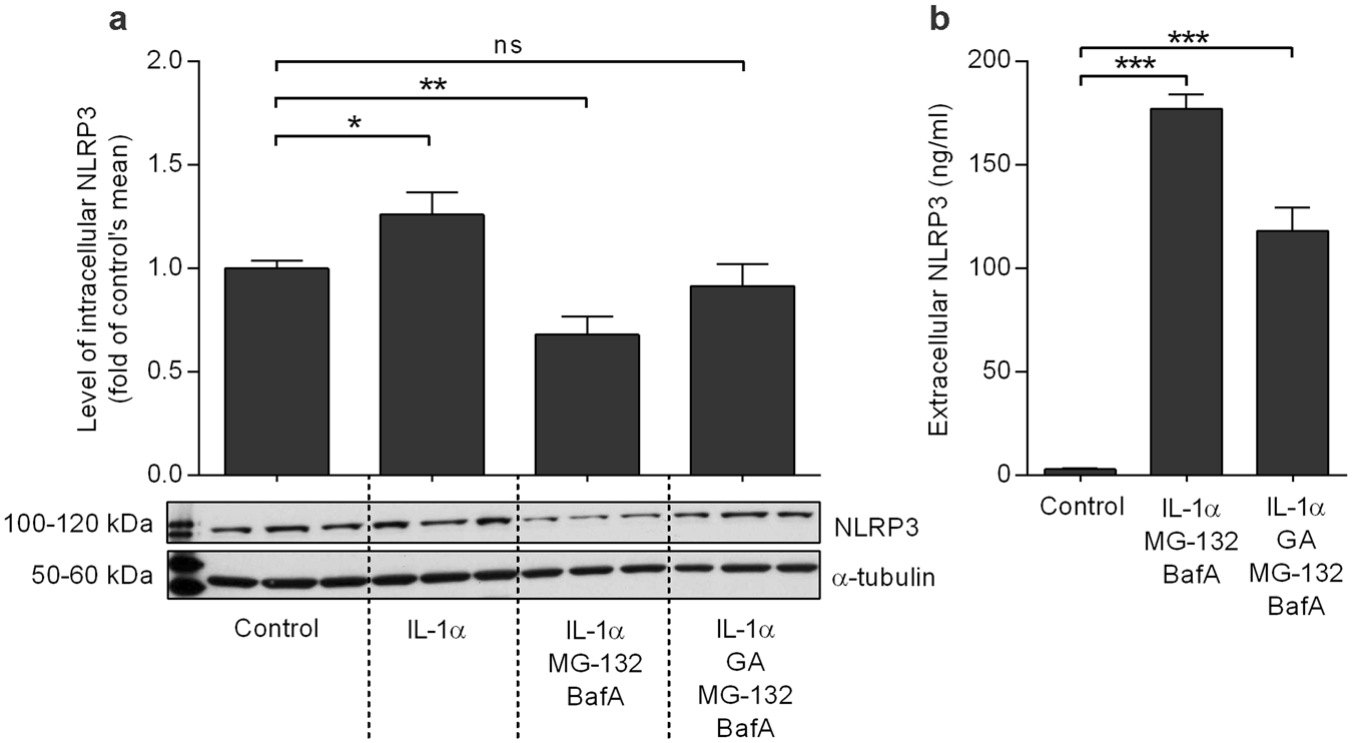
NLRP3 is secreted from the cells when it cannot be degraded by autophagy. The release of NLRP3 is stronger after activation of the NLRP3 inflammasome (concurrent with the release of IL-1β) than after inhibition of inflammasome activation by GA (**a**,**b**). ARPE-19 cells were treated with IL-1α (24 h), geldanamycin (GA) and MG-132 (24 h), and bafilomycin A1 (BafA; 24 h), and both medium and cell lysate samples were collected. The intracellular NLRP3 concentration was measured from cell lysates using the western blot technique, and a representative blot picture is shown (**a**). The extracellular NLRP3 levels were measured from cell culture medium samples using the ELISA technique (**b**). Bars are presented as mean ± SEM. *P < 0.05, **P < 0.01, ***P < 0.001, ns = not significant, Mann –Whitney *U*-test.

It is known that the inflammasome components can be secreted from cells in conjunction with the cytokines, a process which is considered to prevent apoptosis^24–30^. It is not known whether this phenomenon occurs in retinal cells. The priming of ARPE-19 cells with IL-1α significantly up-regulated the protein levels of NLRP3 inside the cells (Fig. 5a). However, when inflammasome signaling was activated by the subsequent exposure of cells to MG-132 + BafA, the intracellular NLRP3 levels were significantly reduced (*P* < 0.01, Fig. 5a). At the same time, there was a concurrent increase in the levels of extracellular NLRP3 (*P* < 0.0001; Fig. 5b). Interestingly, the levels of extracellular NLRP3 were significantly increased also in the presence of GA, which prevented the inflammasome activation (P < 0.0001; Fig. 5b). Intracellular NLRP3 levels were reduced to control levels (Fig. 5a). These results suggest that RPE cells remove activated NLRP3 by secretion, and that the same phenomenon also applies to excess levels of the inactive receptor if it cannot be degraded via autophagy. The release of inactive NLRP3 is more moderate when compared to that of the activated receptor but still significantly higher when compared to control.

## Discussion

In age-related macular degeneration, destruction of the RPE layer results in the death of foveal cone photoreceptors that are responsible for the sharp central vision^31^. This means that RPE cells are critical in the development of AMD pathology. Inflammasome signaling has recently been associated with the pathogenesis of AMD^32–34^. This pathway results in the maturation of IL-1β, which we have previously shown to predominate in our aggregation model of AMD^18^. The release of inflammasome-related cytokines is highly dependent on both the stimulus and the cell type^35^. We and others have shown that ARPE-19 cells are a poor producer of IL-1β. However, we have repeatedly demonstrated the activation of the inflammasome in ARPE-19 cells, showing that these cells can serve as a flexible tool enabling extensive screening protocols^18,34^. In the present study, we confirmed the functionality of our aggregation model by demonstrating that the effects previously seen in ARPE-19 cells were duplicated in hESC-RPE and primary hRPE cells. Although inflammasome activation can be triggered by combined MG-132 + BafA treatment^18^, our present data showed that priming with IL-1α enhances that response even more. Our data is in line with previous results that IL-1α is capable of inducing pro-IL-1β expression in ARPE-19 cells^3^. IL-1α is also a much more potent primer of the NLRP3 inflammasome in ARPE-19 cells than LPS, which we used in our previous study with 4-hydroxynonenal^34^.

Heat shock proteins (Hsps) are chaperones assisting the folding of newly synthetized proteins, repairing misfolded proteins, and preventing harmful aggregation^36^. Hsps 27 and 70 are inducible proteins, whereas Hsp60, Hsc70, and Hsp90 are continuously expressed in mammalian cells^37^. If NLRP3 is translated after the priming signal, it is removed from the cell unless protected by a protein complex containing Hsp90^14–16^. Mayor *et al*. showed that in the absence of Hsp90, NLRP3 becomes degraded by the proteasome^14^. In agreement with their findings, in our study, GA reduced the inflammasome-related response, indicating that NLRP3 would be removed without the protection proffered by Hsp90. The levels of NLRP3 remained reduced irrespective of the addition of GA before or after the proteasomal inhibitor MG-132 in RPE cells, but were unaffected if GA was added after autophagy inhibition. This implies that NLRP3 is degraded also when proteasomes are not working as long as autophagy is functional. Our study differs from the report by Mayor *et al*. mainly in that their cells were monocytes and macrophages. The cell type can indeed affect the outcome, evidence that different tissues and cells throughout the body may have their own distinctive reactions to treatment.

As reviewed by Harris *et al*.^38^. and observed also by ourselves, autophagy and inflammasomes regulate each other, making it most likely that autophagy could be responsible for the removal of unnecessary inflammasome components. Autophagy is the ultimate cellular degradation system, especially when proteasomal degradation has failed. It is known that proteasomal activity decreases during aging; this process has also been associated with the pathogenesis of AMD^39,40^. Since autophagy is responsible for the degradation of large protein aggregates, it could also participate in the removal of inflammasome complexes after activation, as long as it is functional. In the aged RPE, material destined for elimination may also be transported by exocytosis. This concept has been considered as a potential mechanism to explain the formation of drusen material deposited between the RPE cells and the Bruch’s membrane^41^. Our present data is in line with the studies showing that exocytosis can be an alternative and supplementary method for removing NLRP3 released from its protecting complex. Numerous studies on different cell types have shown that inflammasome components can be secreted from the cell following their activation^24–30^. This process is considered to prevent cell death, but it has not yet been demonstrated in RPE cells. Already in their seminal publication where Martinon *et al*. introduced the inflammasome, authors suggested that the whole inflammasome complex can be secreted out of the cell along with active IL-1β^24^. That work had been performed using LPS-treated THP-1 macrophages. The release of inflammasome components was confirmed by Mariathasan *et al*. using primary macrophages exposed to the intracellular pathogen Salmonella typhimurium^25^. Thereafter, inflammasome components have been shown to be secreted also from UVB-induced keratinocytes^27^ and IMR90 cells undergoing oncogene-induced senescence (OIS)^29^. Keller *et al*. showed on activated macrophages and UV-treated keratinocytes that active caspase-1 promotes its own secretion along with its substrates^28^. Oligomeric inflammasome particles released by macrophages have also been shown to function as danger signals and spread inflammation to surrounding cells^30^. Our recent data suggest that especially if NLRP3 remains non-degraded due to invalid autophagy, it can be released also from RPE cells. It is obvious that NLRP3 does not leak passively from the cells since the levels of LDH decreased concurrently with the increased IL-1β secretion. Although it is evident that MG-132 + BafA causes some damage to the plasma membrane, reflected in the increased release of LDH, it is important to note that our IL-1β ELISA kit measures the mature and not the pro-form of the cytokine, as we have previously demonstrated^18^. Furthermore, the significant differences between the levels of IL-1β in medium vs. cell lysate^18^ support the view that MG-132 + BafA-treated cells are functioning actively rather than simply passively releasing their contents. Furthermore, neither the caspase-1 inhibitor nor GA could prevent the toxicity caused by MG-132 + BafA but still there are significant differences in the released cytokine levels, which would be non-existent if cell membranes had simply been ruptured. All of these findings support the concept that the release of NLRP3 is intentional and not attributable to passive leakage.

Hsp90 inhibitors have been developed and tested as drugs, especially for treating cancers. Aberrant chaperone ligands under pathogenic conditions have been implicated also in many neurodegenerative and aggregation diseases, making Hsp90 inhibitors also an interesting therapy option for them^42^. Hsp90 in the extracellular space of RPE induces inflammation, which can be prevented by inhibitors^43^. Geldanamycin and its synthetic derivative 17-AAG have been shown to induce Hsp70 expression as well as suppressing protein aggregation and inhibiting hypoxia-induced production of factors contributing to neovascularization in RPE cells^44–47^. All of those properties would enhance cell survival and combat the pathogenesis of AMD. 17-AAG exerted beneficial effects also in murine models of endotoxin-induced uveitis, retinitis pigmentosa, and inherited retinal degeneration^48–50^. Despite these positive findings, many trials have suffered from adverse effects including ocular toxicity accompanied by visual disturbances^51–54^. Our present data reveal a new mechanism and indicate that the regulation of Hsp90 would be efficient in reducing inflammasome activation in the RPE. The agent used in our studies, GA, is too toxic to be administered to humans and some other entities will need to be tested in subsequent studies. One possibility could be the 4-(1H-pyrazolo[3,4-b]pyridine-1-yl)benzamide TAS-116 developed by Japanese researchers. TAS-116 is a selective inhibitor of cytosolic Hsp90^55^. Unlike 17-AAG, 17-DMAG, NVP-AUY922, BIIB021, or SNX-2112, TAS-116 does not inhibit other Hsp90 paralogs, such as GRP94 in the endoplasmic reticulum or TRAP1 in mitochondria^55^. In rats, orally administered TAS-116 did not cause photoreceptor damage, and it appeared to be well tolerated also by human RPE cells^55^.

**Figure 6:**
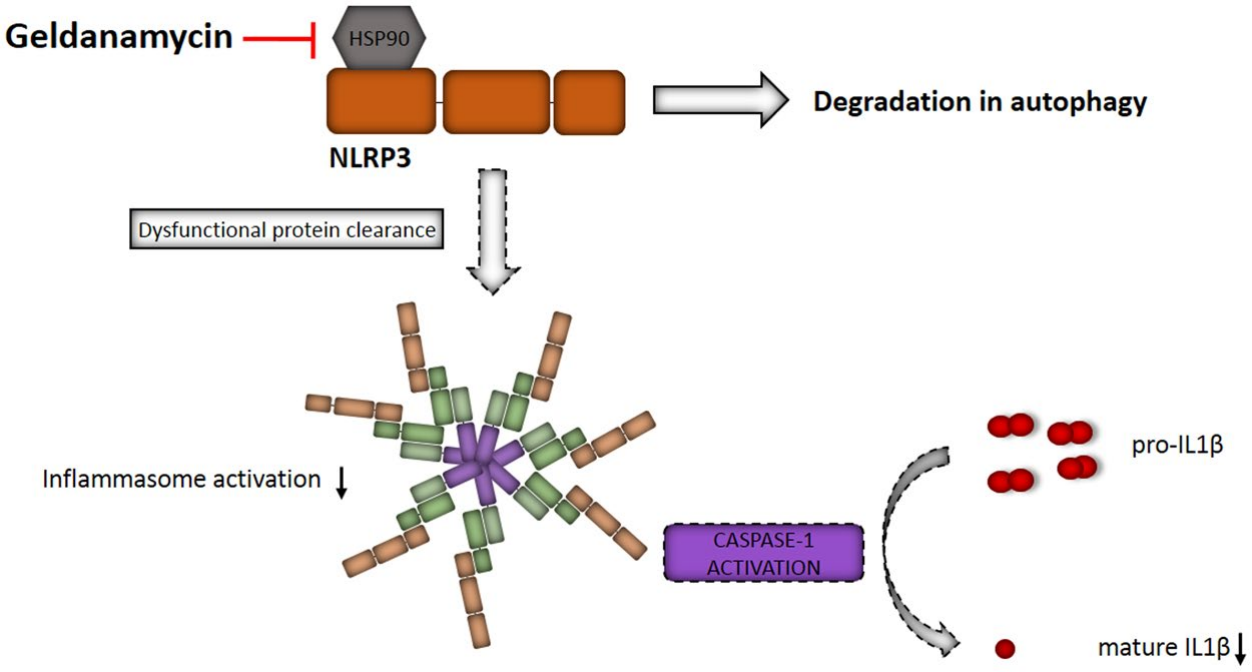
Summary of the effects of geldanamycin on inflammasome activation in human RPE cells. Inhibition of Hsp90 using geldanamycin led to the degradation of NLRP3 by autophagy, which prevented NLRP3 inflammasome signaling and reduced the secretion of mature IL-1β.

We have shown here that the blockade of intracellular degradation systems serves as the inflammasome activator in hESC-RPE and primary hRPE cells, similar to the situation present in ARPE-19 cells. In this improved protocol, the priming phase has been consolidated by supplementation with IL-1α. Moreover, the Hsp90 inhibitor GA appeared capable of preventing the NLRP3 inflammasome activation in human RPE cells. NLRP3 released from the Hsp90 complex prior to activation was removed by autophagy rather than via proteasomal degradation (Fig. 6). The receptor protein was secreted from the cell following its activation, and was partially secreted also in its inactive form when it could not be degraded by autophagy. Together, these data increase the knowledge on the intracellular receptor-mediated processes critical for the inflammasome activation related to the RPE degeneration and its potential as a therapeutic target. Due to its high toxicity, GA cannot be used in clinical settings but these studies need to proceed by testing other NLRP3 regulators e.g. less toxic Hsp90 inhibitors.

## Electronic supplementary material

Supplementary Figure 1.

## References

[1] Martinon F, Mayor A, Tschopp J (2009). The inflammasomes: guardians of the body. Annu. Rev. Immunol..

[2] Sutterwala FS, Haasken S, Cassel SL (2014). Mechanism of NLRP3 inflammasome activation. Ann. N. Y. Acad. Sci..

[3] Tseng WA (2013). NLRP3 inflammasome activation in retinal pigment epithelial cells by lysosomal destabilization: implications for age-related macular degeneration. Invest. Ophthalmol. Vis. Sci..

[4] Jo EK, Kim JK, Shin DM, Sasakawa C (2016). Molecular mechanisms regulating NLRP3 inflammasome activation. Cell. Mol. Immunol..

[5] Petrovski G (2011). Phagocytosis of cells dying through autophagy induces inflammasome activation and IL-1beta release in human macrophages. Autophagy.

[6] Ayna G (2012). ATP release from dying autophagic cells and their phagocytosis are crucial for inflammasome activation in macrophages. PLoS One.

[7] Strowig T, Henao-Mejia J, Elinav E, Flavell R (2012). Inflammasomes in health and disease. Nature.

[8] Celkova L, Doyle SL, Campbell M (2015). NLRP3 Inflammasome and Pathobiology in AMD. J. Clin. Med..

[9] Kauppinen A, Paterno JJ, Blasiak J, Salminen A, Kaarniranta K (2016). Inflammation and its role in age-related macular degeneration. Cell Mol. Life Sci..

[10] Wong WL (2014). Global prevalence of age-related macular degeneration and disease burden projection for 2020 and 2040: a systematic review and meta-analysis. Lancet Glob. Health..

[11] Velez-Montoya R (2014). Current knowledge and trends in age-related macular degeneration: genetics, epidemiology, and prevention. Retina.

[12] Buschini E (2015). Recent developments in the management of dry age-related macular degeneration. Clin. Ophthalmol..

[13] Taipale M, Jarosz DF, Lindquist S (2010). HSP90 at the hub of protein homeostasis: emerging mechanistic insights. Nat. Rev. Mol. Cell Biol..

[14] Mayor A, Martinon F, De Smedt T, Petrilli V, Tschopp J (2007). A crucial function of SGT1 and HSP90 in inflammasome activity links mammalian and plant innate immune responses. Nat. Immunol..

[15] Martinon F, Gaide O, Petrilli V, Mayor A, Tschopp J (2007). NALP inflammasomes: a central role in innate immunity. Semin. Immunopathol..

[16] Martinon F (2008). Detection of immune danger signals by NALP3. J. Leukoc. Biol..

[17] Harris J (2011). Autophagy controls IL-1beta secretion by targeting pro-IL-1beta for degradation. J. Biol. Chem..

[18] Piippo N (2014). Decline in cellular clearance systems induces inflammasome signaling in human ARPE-19 cells. Biochim. Biophys. Acta.

[19] Skottman H (2010). Derivation and characterization of three new human embryonic stem cell lines in Finland. In Vitro Cell. Dev. Biol. Anim..

[20] Vaajasaari H (2011). Toward the defined and xeno-free differentiation of functional human pluripotent stem cell-derived retinal pigment epithelial cells. Mol. Vis..

[21] Szatmari-Toth M (2016). Clearance of autophagy-associated dying retinal pigment epithelial cells - a possible source for inflammation in age-related macular degeneration. Cell. Death Dis..

[22] Bradford MM (1976). A rapid and sensitive method for the quantitation of microgram quantities of protein utilizing the principle of protein-dye binding. Anal. Biochem..

[23] Curtis MJ (2015). Experimental design and analysis and their reporting: new guidance for publication in BJP. Br. J. Pharmacol..

[24] Martinon F, Burns K, Tschopp J (2002). The inflammasome: a molecular platform triggering activation of inflammatory caspases and processing of proIL-beta. Mol. Cell.

[25] Mariathasan S (2004). Differential activation of the inflammasome by caspase-1 adaptors ASC and Ipaf. Nature.

[26] Martinon F, Tschopp J (2004). Inflammatory caspases: linking an intracellular innate immune system to autoinflammatory diseases. Cell.

[27] Feldmeyer L (2007). The inflammasome mediates UVB-induced activation and secretion of interleukin-1beta by keratinocytes. Curr. Biol..

[28] Keller M, Ruegg A, Werner S, Beer HD (2008). Active caspase-1 is a regulator of unconventional protein secretion. Cell.

[29] Acosta JC (2013). A complex secretory program orchestrated by the inflammasome controls paracrine senescence. Nat. Cell Biol..

[30] Baroja-Mazo A (2014). The NLRP3 inflammasome is released as a particulate danger signal that amplifies the inflammatory response. Nat. Immunol..

[31] Kaarniranta K (2013). Autophagy and heterophagy dysregulation leads to retinal pigment epithelium dysfunction and development of age-related macular degeneration. Autophagy.

[32] Tarallo V (2012). DICER1 loss and Alu RNA induce age-related macular degeneration via the NLRP3 inflammasome and MyD88. Cell.

[33] Doyle SL (2012). NLRP3 has a protective role in age-related macular degeneration through the induction of IL-18 by drusen components. Nat. Med..

[34] Kauppinen A (2012). Oxidative stress activates NLRP3 inflammasomes in ARPE-19 cells–implications for age-related macular degeneration (AMD). Immunol. Lett..

[35] Shi G (2015). Inflammasomes Induced by 7-Ketocholesterol and Other Stimuli in RPE and in Bone Marrow-Derived Cells Differ Markedly in Their Production of IL-1beta and IL-18. Invest. Ophthalmol. Vis. Sci..

[36] Lamark T, Johansen T (2012). Aggrephagy: selective disposal of protein aggregates by macroautophagy. Int. J. Cell. Biol..

[37] Park JW (2007). Differential expression of heat shock protein mRNAs under *in vivo* glutathione depletion in the mouse retina. Neurosci. Lett..

[38] Harris H, Rubinsztein DC (2011). Control of autophagy as a therapy for neurodegenerative disease. Nat. Rev. Neurol..

[39] Li Y (2008). Alterations of activity and intracellular distribution of the 20S proteasome in ageing retinal pigment epithelial cells. Exp. Gerontol..

[40] Jung T, Catalgol B, Grune T (2009). The proteasomal system. Mol. Aspects Med..

[41] Kinnunen K, Petrovski G, Moe MC, Berta A, Kaarniranta K (2012). Molecular mechanisms of retinal pigment epithelium damage and development of age-related macular degeneration. Acta Ophthalmol..

[42] Carman A, Kishinevsky S, Koren J, Lou W, Chiosis G (2013). Chaperone-dependent Neurodegeneration: A Molecular Perspective on Therapeutic Intervention. J. Alzheimers Dis. Parkinsonism.

[43] Qin S (2011). Inhibition of RPE cell sterile inflammatory responses and endotoxin-induced uveitis by a cell-impermeable HSP90 inhibitor. Exp. Eye Res..

[44] Kaarniranta K (2005). Geldanamycin activates Hsp70 response and attenuates okadaic acid-induced cytotoxicity in human retinal pigment epithelial cells. Brain Res. Mol. Brain Res..

[45] Wu WC, Kao YH, Hu PS, Chen JH (2007). Geldanamycin, a HSP90 inhibitor, attenuates the hypoxia-induced vascular endothelial growth factor expression in retinal pigment epithelium cells *in vitro*. Exp. Eye Res..

[46] Wang YQ, Zhang XM, Wang XD, Wang BJ, Wang W (2010). 17-AAG, a Hsp90 inhibitor, attenuates the hypoxia-induced expression of SDF-1alpha and ILK in mouse RPE cells. Mol. Biol. Rep..

[47] Ryhanen T (2011). Influence of Hsp90 and HDAC inhibition and tubulin acetylation on perinuclear protein aggregation in human retinal pigment epithelial cells. J. Biomed. Biotechnol..

[48] Poulaki V (2007). Inhibition of Hsp90 attenuates inflammation in endotoxin-induced uveitis. FASEB J..

[49] Tam LC (2010). Prevention of autosomal dominant retinitis pigmentosa by systemic drug therapy targeting heat shock protein 90 (Hsp90). Hum. Mol. Genet..

[50] Aguila M (2014). Hsp90 inhibition protects against inherited retinal degeneration. Hum. Mol. Genet..

[51] Kummar S (2010). Phase I trial of 17-dimethylaminoethylamino-17-demethoxygeldanamycin (17-DMAG), a heat shock protein inhibitor, administered twice weekly in patients with advanced malignancies. Eur. J. Cancer.

[52] Pacey S (2011). A phase I study of the heat shock protein 90 inhibitor alvespimycin (17-DMAG) given intravenously to patients with advanced solid tumors. Clin. Cancer Res..

[53] Sessa C (2013). First-in-human phase I dose-escalation study of the HSP90 inhibitor AUY922 in patients with advanced solid tumors. Clin. Cancer Res..

[54] Shapiro GI (2015). First-in-human phase I dose escalation study of a second-generation non-ansamycin HSP90 inhibitor, AT13387, in patients with advanced solid tumors. Clin. Cancer Res..

[55] Ohkubo S (2015). TAS-116, a highly selective inhibitor of heat shock protein 90alpha and beta, demonstrates potent antitumor activity and minimal ocular toxicity in preclinical models. Mol. Cancer. Ther..

