# Supplementary material for: Hsp90 inhibition as a means to inhibit activation of the NLRP3 inflammasome
Source: Sci Rep. 2018 Apr 30;8:6720. doi: 10.1038/s41598-018-25123-2 (PMC5928092; doi:10.1038/s41598-018-25123-2)
Supplement: Supplementary file 1 — Supplementary Figure 1. [file 41598_2018_25123_MOESM1_ESM.pdf]

Hsp90 inhibition as a means to inhibit activation of the NLRP3 inflammasome

Niina Piippo[1], Eveliina Korhonen[1], Maria Hytti[1], Heli Skottman[2], Kati Kinnunen[3], Natasha Josifovska[4], Goran Petrovski [4,5], Kai Kaarniranta[3,6], Anu Kauppinen[1,*]

[1]School of Pharmacy, University of Eastern Finland, Kuopio, 70211, Finland

[2]Faculty of Medicine and Life Sciences, BioMediTech, University of Tampere, Tampere, 33014, Finland

[3]Department of Ophthalmology, Kuopio University Hospital, Kuopio, 70211, Finland

[4]Stem Cells and Eye Research Laboratory, Department of Ophthalmology, Faculty of Medicine, Albert Szent-Györgyi Clinical Center, University of Szeged, Szeged, Hungary

[5]Center for Eye Research, Department of Ophthalmology, Oslo University Hospital, University of Oslo, Oslo, Norway

[6]Department of Ophthalmology, Institute of Clinical Medicine, University of Eastern Finland, Kuopio, 70211, Finland

***Corresponding author:** Anu Kauppinen, School of Pharmacy, Faculty of Health Sciences, University of Eastern Finland, P.O. Box 1627, FIN-70211 Kuopio, Finland, Fax: +358 17 162048, Tel.: +358 40 3553216, e-mail: anu.kauppinen@uef.fi

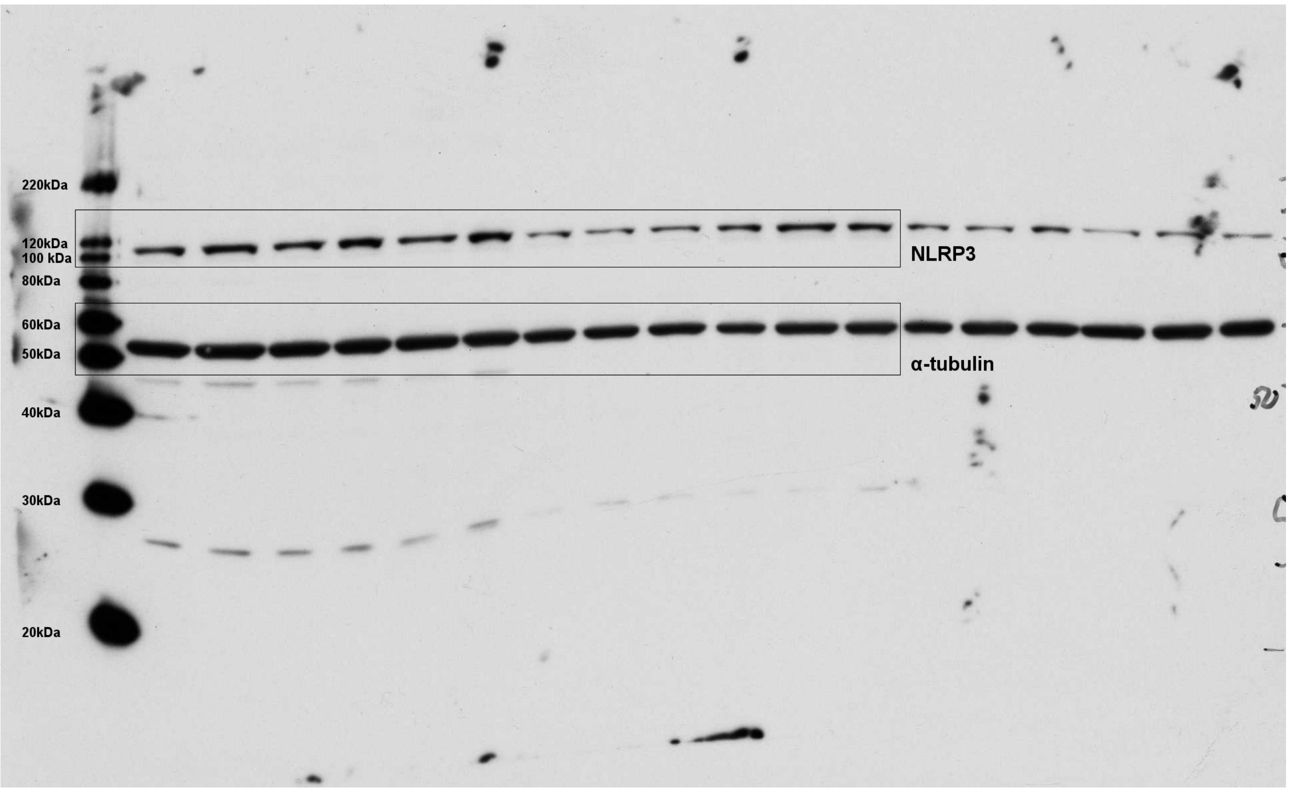

Supplementary Figure 1. Scan of the complete blot represented in Figure 5. NLRP3 and α-tubulin, as an internal control, were detected from the same blot. Boxes indicate the cropped lanes shown in Figure 5.
